# Post‐transcriptional regulation of PIAS3 expression by miR‐18a in malignant mesothelioma

**DOI:** 10.1002/1878-0261.12386

**Published:** 2018-10-23

**Authors:** Tian He, Karen McColl, Nneha Sakre, Yanwen Chen, Gary Wildey, Afshin Dowlati

**Affiliations:** ^1^ Department of Biochemistry School of Medicine, Case Western Reserve University Cleveland OH USA; ^2^ Division of Hematology and Oncology Case Western Reserve University and University Hospitals Seidman Cancer Center Cleveland OH USA; ^3^ Department of Population and Quantitative Health Sciences School of Medicine, Case Western Reserve University Cleveland OH USA

**Keywords:** mesothelioma, microRNA, PIAS3, STAT3

## Abstract

Protein inhibitor of activated STAT3 (PIAS3) is an endogenous suppressor of signal transducer and activator of transcription 3 (STAT3) signaling. By directly interacting with phosphorylated STAT3, PIAS3 can block the downstream transcriptional activity of STAT3, which is hyper‐activated in various cancers. We previously reported that in malignant mesothelioma (MM), low PIAS3 expression is associated with increased STAT3 activation and correlates with poor patient survival, yet the regulatory mechanism(s) governing PIAS3 expression in MM remain unclear. Here, we demonstrate that PIAS3 protein expression does not correlate with its mRNA level in MM cell lines, indicating that PIAS3 expression is regulated at a post‐transcriptional level. Inhibition of proteasomal degradation with MG132 (10 μm) or bortezomib (1 μm), alone and in combination, did not increase PIAS3 protein levels; furthermore, inhibition of protein synthesis by cycloheximide treatment did not decrease PIAS3 levels within 48 h, suggesting that PIAS3 expression is not actively regulated at a post‐translational level. To determine whether miRNA (miRs) can translationally regulate PIAS3 expression, we combined miR microarray analysis with bioinformatic screening to identify candidate miRs, in MM cell lines with low PIAS3 expression, followed by luciferase reporter assays to validate miR regulation of the PIAS3 3′UTR. We identified miR‐18a as a suppressor of PIAS3 expression that is upregulated in MM cells and whose inhibition can increase PIAS3 expression and suppress STAT3 activity. Moreover, we showed that miR‐18a inhibition can decrease MM cell viability and that its expression is negatively correlated with MM patient survival. Taken together, these results suggest that targeting miR‐18a may have therapeutic benefit in MM.

**Abbreviations**
3′UTR: 3′ untranslated region
miR: microRNA
MM: malignance mesothelioma
PIAS3: protein inhibitor of activated STAT3
STAT3: signal transducer and activator of transcription 3

## 1 Introduction

PIAS3 (protein inhibitor of activated STAT3) was originally identified as a major negative regulator of STAT3 (signal transducer and activator of transcription 3) activity (Chung *et al*., 1997). STAT3 is a key transcriptional regulator of diverse cellular activities, including proliferation, differentiation, metabolism, and apoptosis. When stimulated by its upstream activators, that is, growth factors and cytokines, STAT3 is phosphorylated, dimerizes into its active form, and then enters the nucleus and binds to DNA to regulate gene transcription (Al Zaid Siddiquee and Turkson, 2008; Shuai and Liu, 2003; Yu and Jove, 2004). PIAS3 can directly bind to activated STAT3 (p‐STAT3) and suppress its transcriptional activity (Chung *et al*., 1997). We have validated this model in lung cancer cell lines, demonstrating that once STAT3 is activated by upstream epidermal growth factor stimulation, PIAS3 is rapidly translocated from the cytoplasm into the nucleus and associates with p‐STAT3 to form a complex and downregulate STAT3 transcriptional activity (Dabir *et al*., 2009).

To date, PIAS3 expression has been found to be reduced in multiple malignancies, including gastric cancer, glioblastoma, ovarian cancer, and breast cancer (Borghouts *et al*., 2010; Brantley *et al*., 2008; Saydmohammed *et al*., 2010). Our previous findings have shown that PIAS3 expression is downregulated in lung squamous cell cancer and malignant mesothelioma (MM) (Abbas *et al*., 2015; Dabir *et al*., 2014). Furthermore, we and others have shown that downregulated PIAS3 expression in tumors is correlated with hyper‐active STAT3 signaling, increased proliferation, survival, and migration in cancer cell lines and poor patient overall survival in the clinic (Abbas *et al*., 2015; Brantley *et al*., 2008; Dabir *et al*., 2014), suggesting impaired PIAS3 regulation of activated STAT3 in these cancers. Studies have also indicated that restoring or overexpressing PIAS3, via repression of STAT3‐mediated oncogenic pathways, shows inhibitory effects on cancer cells (Borghouts *et al*., 2010; Brantley *et al*., 2008; Dabir *et al*., 2014; Saydmohammed *et al*., 2010). All this evidence suggests that PIAS3 plays an important role as a tumor suppressor in many cancers.

MicroRNA (miRNA) are an important class of post‐transcriptional regulators that have been discovered in recent years that are noncoding small RNA 19–22 nucleotides in length (Pichler and Calin, 2015; Ventura and Jacks, 2009). By imperfectly base pairing to the 3′UTR of target messenger RNA (mRNA), miRNA can cause mRNA degradation or translational silencing and eventually repress gene expression (Pichler and Calin, 2015; Ventura and Jacks, 2009). Alterations in post‐transcriptional regulation by miRNA have been described in different pathological conditions, and in the context of cancer, where miRNA can potentiate or attenuate tumorigenesis by affecting expression of various oncogenes or tumor suppressors, deregulation of miRNA is often a driving factor in cancer development and progression. In fact, a wide variety of upregulated miRNA in cancer have been identified as ‘oncomiRs’ due to their close association with oncogenesis (Pichler and Calin, 2015; Ventura and Jacks, 2009).

In this study, we explored the regulation of PIAS3 expression in MM. MM is a highly‐aggressive thoracic cancer with poor prognosis and limited therapeutic options (Kim *et al*., 2017). Genomic studies have revealed that MM is characterized by frequent mutations in tumor suppressor genes (Yap *et al*., 2017), making the study of PIAS3 in this cancer highly relevant. Because of the long latency to diagnosis following exposure to asbestos, the principal carcinogen, and an increasing global incidence rate, there is an urgent need to develop more therapeutic targets for MM. Here, we investigated PIAS3 at two different levels: post‐translational regulation by protein degradation and translational regulation by miRNA. We identify miR‐18a as a candidate suppressor of PIAS3 expression as well as a potential therapeutic target in MM.

## 2 Materials and methods

### 2.1 Cell lines and tissue culture

Four mesothelioma cell lines (H28, 211H, H2052, and H2452) and one nonsmall cell lung cancer (NSCLC) cell line (A549) were purchased from ATCC (Manassas, VA, USA) and cultured in DMEM/F12 with 10% FBS and 5% Glutamax. HEK293T cells were cultured in Dulbecco's modified Eagle's medium with 10% FBS and 5% Glutamax. Two more mesothelioma cell lines (HAY and YOU) were kindly provided by R. Hassan from the National Cancer Institute and were cultured in RPMI‐1640 with 10% FBS, 5% Glutamax and 5% sodium pyruvate.

### 2.2 Western blotting

Protein lysates were prepared and analyzed on 4–20% Criterion gels (Bio‐Rad, Hercules, CA, USA) as described previously (Unkila *et al*., 2001). Primary antibodies used were as follows: PIAS3 (Cell Signaling Technology, Danvers, MA, USA, #4164), TP53 (Santa Cruz Biotechnology, Dallas, TX, USA, sc‐71817), survivin (Cell Signaling Technology #2803), and beta‐actin (Sigma, St. Louis, MO, USA, A‐5441).

### 2.3 Real‐time quantitative RT‐PCR

Cell lines were harvested, and total RNA was isolated using TRIzol (Invitrogen) and miRNA using a miRNeasy Mini Kit (QIAGEN, Hilden, Germany) following manufacturer's instructions. For PIAS3 RT‐qPCR, cDNA synthesis was conducted using the High‐capacity cDNA reverse transcription kit (Applied Biosystems). For miR RT‐qPCR, cDNA synthesis was conducted using the Taqman miRNA reverse transcription kit (Thermofisher Scientific). Real‐time quantitative PCR was performed by a Lightcycler 480 II (Roche, Indianapolis, IN, USA) using TaqMan probe primer mixes for PIAS3 or for miR‐335‐5p, miR‐543‐5p, miR‐31‐5p, and miR‐18a‐5p (Thermofisher Scientific). Results were normalized to housekeeping gene levels: beta‐actin for PIAS3 mRNA and miR‐16/RNU6B for miRs.

### 2.4 Proteasome inhibitor and cycloheximide treatment of cell lines

The proteasome inhibitors MG‐132 (ENZO Lifescience, Farmingdale, NY, USA) and bortezomib (Selleck, Houston, TX, USA) and the protein synthesis inhibitor cycloheximide (CHX) (ENZO Lifescience) were dissolved in DMSO to prepare stock solutions and then diluted in cell culture medium to their final concentrations. Three MM cell lines with low PIAS3 expression (211H, H2052, and H2452) were seeded into 100 mm tissue culture plates for overnight incubation, and the next day, cells were treated with MG‐132 at 10 μm or with bortezomib at 1 μm, separately or in combination, for 5 h. The H28 MM cell line with high PIAS3 protein expression was seeded into 100 mm tissue culture plates for overnight incubation, and the next day, cells were treated with CHX at 100 μg·mL^−1^ for 4, 8, 24, and 48 h. After treatment, cells were harvested and lysed for western blot analysis and comparisons made to DMSO vehicle controls.

### 2.5 miRNA microarray profiling in cell lines

For each cell line, miRNA were isolated using a miRNeasy Mini Kit (QIAGEN), and expression analyzed using miRNA 4.0 Arrays (Affymetrix, Santa Clara, CA, USA), which includes probes for 2578 human mature miRNA and 2025 human pre‐miRNA. Microarray data analysis and expression calculations were performed by the Gene Expression Core facility of the Case Comprehensive Cancer Center. miR candidates were initially selected if they were upregulated in at least one of the MM cells having low PIAS3 protein expression (211H, H2052, and H2452) relative to H28 cells that demonstrate high PIAS3 protein expression. If a miR candidate was upregulated in more than one PIAS3‐deficient MM cell line, then the average upregulation was used to calculate fold difference over H28 cell levels.

### 2.6 3′UTR luciferase reporter assays

Human PIAS3 3′UTR luciferase plasmid (LightSwitch 3′UTR Reporter plasmid) was purchased from Switchgear Genomics (Carlsbad, CA, USA). Specifically, the plasmid contains the PIAS3 3′UTR downstream of a Renilla luciferase coding sequence. To investigate regulation of the PIAS3 3′UTR by different miRs, HEK293T cells were seeded into a 96‐well plate for overnight incubation, then co‐transfected with 100 ng of the PIAS3 3′UTR luciferase plasmid (Switchgear Genomics) per well along with either miR mimics (QIAGEN) or nontargeting control RNAi (QIAGEN) using Lipofectamine 2000 (Invitrogen) following manufacturer's instructions. After 24 h of transfection, the cells were lysed and assayed using a Switchgear Genomics luciferase kit according to the manufacturer's instructions. To further validate the binding between PIAS3 3′UTR and miR‐18a, HEK293T cells were co‐transfected with 100 ng PIAS3 3′UTR plasmid along with miR‐18a or scramble control at three different concentrations (10, 30 and 50 nM); cells were lysed and assayed 24 h after the co‐transfection.

For dual‐luciferase reporter assays, human PIAS3 3′UTR or beta‐actin 3′UTR was cloned downstream of a firefly luciferase coding sequence in pmirGLO Dual‐Luciferase miRNA Target Expression Vector (Promega, Madison, WI, USA), which also contains a constitutively driven Renilla luciferase coding sequence for transfection efficiency normalization. Similar to above, cells were seeded into a 96‐well plate and co‐transfected with 100 ng PIAS3 3′UTR dual‐luciferase plasmid and either miR‐18a mimics or scramble control. As a control, co‐transfections were also conducted using an ‘empty’ dual‐luciferase plasmid with a minimal 3′UTR. Twenty‐four hours after the co‐transfection, cells were lysed and assayed using the Dual‐Luciferase Reporter Assay System (Promega).

### 2.7 miR‐18a inhibition assays

For miR‐18a inhibition, electroporation was used to transiently transfect miScript miRNA‐18a inhibitors (QIAGEN) at 100 nm into H2452 MM cells, in parallel with mock electroporation as a control; 48 and 72 h after the transfection, cells were harvested for both RNA and protein extraction and analysis.

To measure STAT3 activity after miR‐18a inhibition, a STAT3‐luciferase reporter assay was used in which firefly luciferase was driven by a sis‐inducible response element (SIE) responsive to STAT3 levels (Promega). H2052 cells were seeded into a 96‐well plate, and Lipofectamine 2000 was used to co‐transfect cells with 100 ng STAT3 luciferase plasmid and 50 ng constitutively expressed Renilla luciferase plasmid (for normalization of transfection efficiency) in the presence or absence of 100 nm miR‐18a inhibitors; 24 h after co‐transfection, cells were lysed and assayed using a Dual‐Luciferase Reporter Assay System (Promega).

To determine cell viability after miR‐18a inhibition, H2452 cells were seeded into a 96‐well plate and transfected with 100 nm miR‐18a inhibitors using Lipofectamine 2000. Viable cells in culture were measured using CellTiter‐Glo Luminescence Cell Viability Assay (Promega) at 24, 72, and 100 h after miR‐18a inhibitors transfection.

### 2.8 TCGA survival analysis

The MM miR gene expression data and clinical data were downloaded from the TCGA website with the platform Affymetrix HT_HGU133A. Gene expression data and matching clinical data were available for 87 patients. The TCGA miR gene expression data were extracted and merged with clinical outcome data for further analysis. We examined the predictive value of various miRs on survival treating individual miR gene expression as a continuous measurement as well as a categorical variable, that is, by collapsing miR gene expression values into two groups: lower 50% (44 patients) and upper 50% (43 patients) from the median value. The Kaplan–Meier method was used to estimate the overall survival, and the difference of survival among groups was examined using a log‐rank test. The Cox proportional hazards model was performed to estimate the predictive value of miR gene expression on survival, and the hazard ratio was obtained from this model. Analyses were performed using sas version 9.3 statistical software (SAS Institute Inc., Cary, NC, USA) or r 2.1.15 (https://www.r-project.org/).

### 2.9 Statistical analysis

For luciferase reporter assays, RT‐qPCR, and CellTiter‐Glo assays, data represent mean ± standard deviation (SD) of the number of experimental replicates indicated in figure legends. Statistical analyses were performed using graphpad prism 6 (La Jolla, CA, USA). *P* values were generated by un‐paired *t*‐test and multiple *t*‐test, as indicated, and *P* values ≤ 0.05 were considered significant.

## 3 Results

### 3.1 PIAS3 protein expression does not correlate with PIAS3 mRNA levels

**Figure 1:**
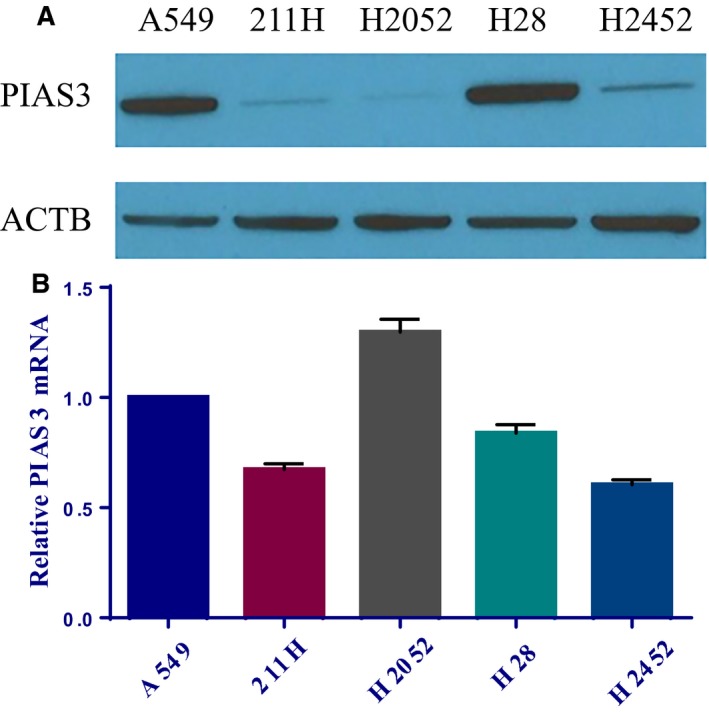
PIAS3 expression in MM cell lines. (A) Western blot of PIAS3 among five cell lines (adenocarcinoma cell line A549; mesothelioma cell lines 211H, H2052, H28, and H2452). (B) Corresponding relative mRNA level of PIAS3 detected by RT‐qPCR, normalized to β‐Actin mRNA. Error bars are representative of Mean ± SD (*n* = 3).

Initially, we investigated endogenous PIAS3 expression in MM cell lines; western blotting and qRT‐PCR were performed to detect PIAS3 protein and mRNA levels, respectively, in four MM cell lines (211H, H2052, H28, H2452); a NSCLC cell line (A549) was used as a positive control (Fig. 1). We found that PIAS3 protein is markedly downregulated in three MM cell lines, 211H, H2052, and H2452, compared to H28 MM cells and A549 cells (Fig. 1A). Western blot of two more MM cell lines (HAY, YOU) also showed low PIAS3 protein levels (Fig. S1). By contrast, PIAS3 mRNA is expressed at a similar level in all five cell lines tested (Fig. 1B). This result indicates that PIAS3 protein expression is not correlated with mRNA levels, suggesting a possible post‐transcriptional regulation of PIAS3 protein expression.

### 3.2 PIAS3 expression is unaffected by proteasome inhibition

**Figure 2:**
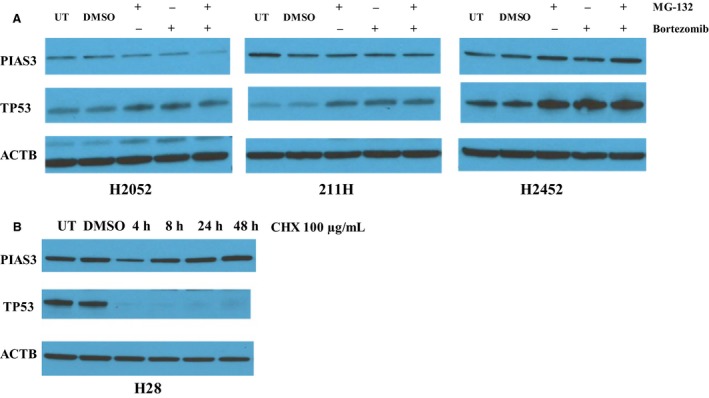
PIAS3 expression after proteasome inhibitor treatment and CHX treatment. (A) Two different proteasome inhibitors, MG‐132 (final concentration of 10 μm) and bortezomib (final concentration of 1 μm), used alone or in combination, were added to mesothelioma cell lines with low PIAS3 expression (211H, H2052, and H2452). After 5 h of treatment, cells were harvested for western blot analysis, with TP53 used as the positive control for proteasome inhibitor efficacy. UT: untreated. DMSO: vehicle added instead of drug. (B) The H28 MM cell line with high PIAS3 protein expression was treated with 100 μg·mL^−1^ CHX for 4, 8, 24, and 48 h. TP53 was used as a positive control for CHX treatment.

To explore whether PIAS3 protein expression is post‐translationally regulated by protein degradation, proteasome inhibitors were incubated with the three MM cell lines with low PIAS3 expression to determine their effect on PIAS3 protein level. As shown in Fig. 2A, while the positive control protein TP53 level was markedly increased by the two different proteasome inhibitors, MG‐132 and bortezomib, alone and in combination, after treatment for 5 h, there was no observed increase in PIAS3 expression. Moreover, CHX treatment of the H28 MM cell line with high PIAS3 protein expression also revealed little degradation of PIAS3 protein (Fig. 2B). These results indicate that protein degradation does not play a significant role in PIAS3 expression in MM cell lines.

### 3.3 Screening miRNA as potential regulators of PIAS3 expression

**Figure 3:**
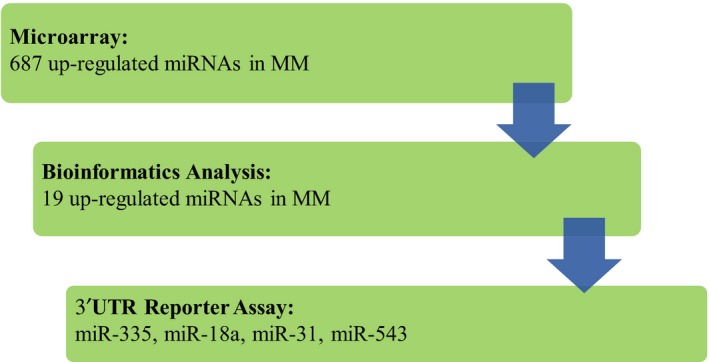
Workflow of miR screen. miR candidates were generated by microarray analysis of MM cell lines followed by bioinformatics analysis (targetscan.org).

miRs are an important class of regulators of protein expression and are frequently dysregulated in cancer. To investigate whether PIAS3 expression is regulated by miRs, we initially used miR microarray analysis of the four MM cell lines to identify miRs that are negatively correlated with PIAS3 expression. Specifically, we looked for miRs that were upregulated in MM cells with low PIAS3 protein levels (211H, H2052, and H2452) relative to H28 cells that demonstrate high PIAS3 protein expression. Using this approach, we identified 687 miR candidates (see Fig. 3, Table S1). We then used TargetScan to identify those candidate miRs with predicted binding sites in the PIAS3 3′UTR. This reduced the number to only 19 candidate miRs (Table 1). Among these remaining PIAS3 miR candidates, miR‐335 and miR‐543 showed the most marked upregulation in MM cell lines with low PIAS3 expression. In addition, miR‐31 has high homology binding to the PIAS3 3′UTR and has been reported as an onco‐miR in lung cancer and is associated with a poor prognosis in MM (Liu *et al*., 2010; Matsumoto *et al*., 2014), although this is controversial (Ivanov *et al*., 2010). These three miRs were chosen as our top candidate regulators of PIAS3 expression along with miR‐18a, which has been reported to be upregulated in both MM cell lines and MM tumor samples compared to normal tissue (Amatya *et al*., 2016; Balatti *et al*., 2011; Ramirez‐Salazar *et al*., 2014). The upregulation of these four miR candidates in MM cell lines was validated by RT‐qPCR (shown in Table 2).

**Table 1:** 19 miR candidates from screening

miR candidate	Average upregulation ratio
miR‐335‐5p	297.53
miR‐543	208.40
miR‐199‐5p	151.15
miR‐154‐5p	46.40
miR‐410‐3p	40.49
miR‐493‐3p	36.02
miR‐31‐5p	20.99
miR‐625‐5p	17.11
miR‐532‐3p	6.43
miR‐328‐3p	5.57
miR‐522‐3p	5.20
miR‐345‐5p	3.81
miR‐3187‐3p	3.49
miR‐361‐3p	2.52
miR‐18a‐5p	1.96
miR‐185‐5p	1.81
miR‐5010‐5p	1.74
miR‐629‐5p	1.61
miR‐186‐5p	1.60

**Table 2:** Validation of microarray results by RT‐qPCR

Normalizing miR	miR‐16			RNU6B		
	211H/H28	H2052/H28	H2452/H28	211H/H28	H2052/H28	H2452/H28
miR‐335	439.7	127.3	11.2	228.1	150.2	23.29
miR‐543	38.3	90.3	0	19.9	106.5	0
miR‐31	73.7	73.1	0	23.1	23.7	0
miR‐18a	3.6	4.2	0.36	1.14	1.46	0.37

Endogenous expression of the four miR candidates (miR‐335, miR‐543, miR‐31, and miR‐18a) in MM cell lines was measured by RT‐qPCR and normalized to two different control miRs: miR‐16 and RNU6B. Candidate miR expression in MM cell lines with low PIAS3 protein levels (211H, H2052, and H2452) was compared to that of the H28 MM cell line demonstrating high PIAS3 protein levels.

### 3.4 miR‐18a can specifically suppress PIAS3 expression at its 3′UTR

**Figure 4:**
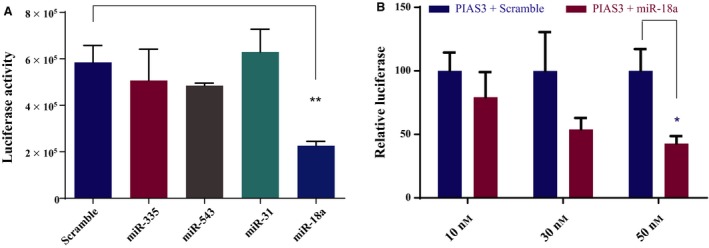
Validation of miR binding to the PIAS3 3′UTR using luciferase reporter assays. (A) Fifty nanomolar of miR mimics (miR‐335, miR‐543, miR‐31, and miR‐18a) or a nontargeting control (scramble) was co‐transfected with 100 ng of PIAS3 3′UTR luciferase plasmid in HEK293T cells and the resulting luciferase activity measured. **P =* *0.0012 comparing miR‐18a to the scramble control. (B) Luciferase reporter assay was performed by co‐transfecting 100 ng PIAS3 3′UTR luciferase plasmid with three different concentrations (10, 30 and 50 nm) of miR‐18a mimics or scramble control in HEK293T cells, and results are normalized to corresponding scramble control. For 50 nm, *P =* *0.0107 comparing miR‐18a to the scramble control. In both (A) and (B), error bars are representative of mean ± SD (*n* = 3). *P* values were calculated using un‐paired *t*‐tests.

To verify the binding of each candidate miR to the PIAS3 3′UTR, reporter assays were performed by co‐transfecting HEK293T cell with miR mimics and a luciferase plasmid containing the human PIAS3 3′UTR. As shown in Fig. 4A, among the four miR candidates, only miR‐18a significantly decreased luciferase activity compared to a nontargeting control RNAi (scramble) (*P *=* *0.0012), demonstrating the specificity of miR‐18a binding to the PIAS3 3′UTR. Furthermore, PIAS3 luciferase activity decreased with increasing amounts of miR‐18a mimics (Fig. 4B), indicating that the binding of miR‐18a to the PIAS3 3′UTR is dose‐dependent.

**Figure 5:**
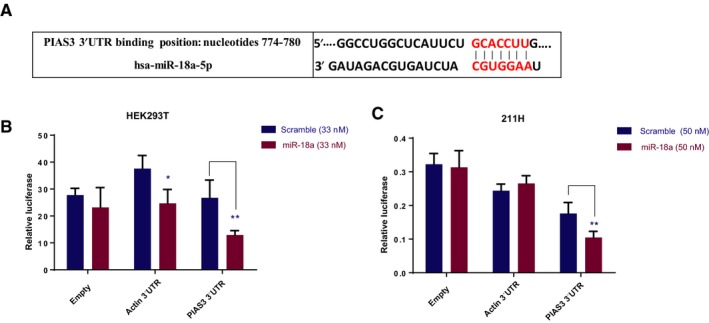
Effect of miR‐18a on dual‐luciferase reporters with different 3′UTRs. (A) Binding site of miR‐18a in the PIAS3 3′UTR predicted from Targetscan.org. (B) Thirty‐three nanomolar of miR‐18a or a nontargeting control (scramble) were co‐transfected with 100 ng of a dual‐luciferase reporter containing a minimal 3′UTR (Empty), the beta‐actin 3′UTR (Actin), or the PIAS3 3′UTR dual‐luciferase plasmid in HEK293T cells. Results are normalized to corresponding scramble control. *P =* *0.011 and **P =* *0.0067 comparing miR‐18a to its matching scramble control. (C) Same as (B) except using the MM cell line 211H and 50 nm of either scramble siRNA or miR‐18a (**P =* *0.009 comparing miR‐18a to its matching scramble control). In (B) and (C), error bars are representative of mean ± SD (*n* = 4). *P* values were calculated using multiple *t*‐test.

To further demonstrate the specificity of miR‐18a binding to its predicted site in the PIAS3 3′UTR, shown in Fig. 5A, we compared the inhibition by miR‐18a mimics on a dual‐luciferase plasmid containing the human PIAS3 3′UTR with that on two control dual‐luciferase plasmids, an ‘empty’ plasmid with a minimal 3′UTR and a beta‐actin 3′UTR plasmid, in HEK293T cells and 211H MM cells. As shown in Fig. 5B,C, compared to scramble control, miR‐18a decreased luciferase activity of the PIAS3 3′UTR plasmid more than it did of the other two control plasmids.

In addition to luciferase expression assays, we also attempted to alter endogenous PIAS3 levels after transient transfection of miR‐18a mimics at 50 nm into the H28 cell line with high endogenous PIAS3. Unfortunately, we could not show a reduction in PIAS3 protein by western blotting after 48 h (data not shown). This is likely related to the high PIAS3 protein stability we previously observed in our CHX assays (Fig. 2B).

### 3.5 Inhibition of endogenous miR‐18a restores PIAS3 expression and suppresses STAT3 activity

**Figure 6:**
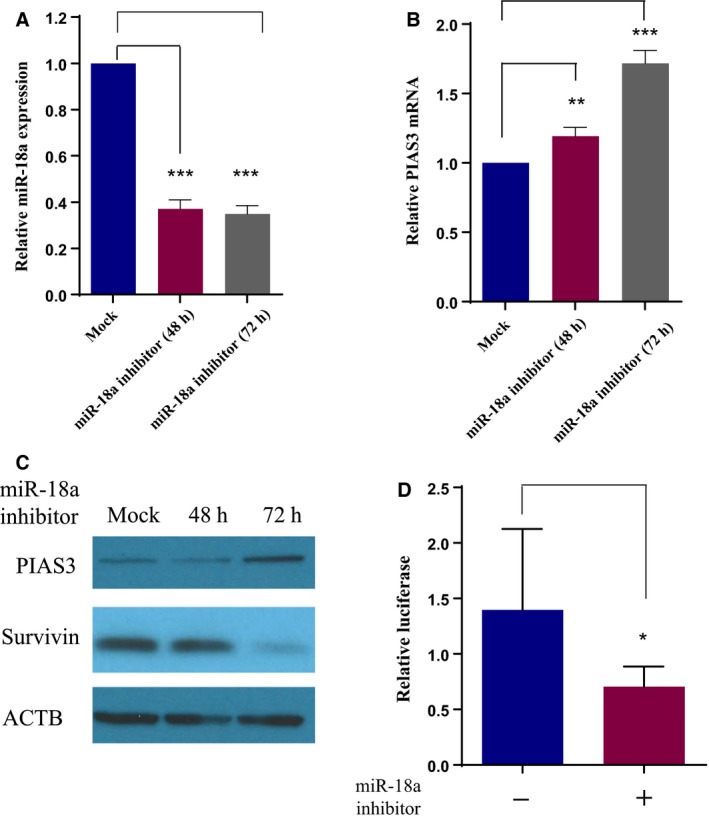
Effect of miR‐18a inhibition by antagomirs. (A) Relative amount of miR‐18a (normalized to RNU6B) 48 and 72 h after transfecting miR‐18a inhibitors at 100 nm into the MM cell line H2452, ***P value < 0.0001 at 48 and 72 h comparing to the mock control. (B) Relative PIAS3 mRNA (normalized by ACTB) detected by qRT‐PCR 48 and 72 h after miR‐18a inhibition in H2452 cells, **P =* *0.0062 at 48 h and ***P =* *0.0002 at 72 h comparing miR‐18a inhibitor group to the mock control. Error bars in (A) and (B) represent Mean ± SD (*n* = 3). (C) Western blot of PIAS3 and survivin in H2452 cells 48 and 72 h after miR‐18a inhibition. (D) p‐STAT3 activity measured by luciferase reporter assay 24 h after transfection with 100 nm miR‐18a inhibitors into H2052 MM cells, error bars stand for Mean ± SD (*n* = 8). *P =* *0.021. All *P* values were calculated using un‐paired *t*‐test.

Finally, we validated the role of miR‐18a in regulating endogenous PIAS3 protein expression by examining the effect of inhibiting miR‐18a in MM cells with low PIAS3 expression. As shown in Fig. 6A, endogenous miR‐18a is significantly decreased by transfecting miR‐18a inhibitor in H2452 MM cells. This inhibition is accompanied by increased PIAS3 expression at both the mRNA (Fig. 6B) and protein level (Fig. 6C) within 72 h after the transfection, suggesting that PIAS3 expression can be restored by blocking endogenous miR‐18a. Moreover, we observed a decreased expression of survivin (Fig. 6C), a downstream target of activated STAT3, indicating that elevating PIAS3 expression by miR‐18a inhibition can suppress STAT3 signaling.

To further investigate downstream effects of elevated PIAS3 expression after miR‐18a inhibition, we performed a STAT3 luciferase reporter assay after transfecting miR‐18a inhibitors into H2052 MM cells (shown in Fig. 6D). Compared to the control group, STAT3 luciferase activity was significantly decreased after miR‐18a inhibition (*P *=* *0.021), suggesting that miR‐18a inhibition suppresses STAT3 transcriptional activity in MM cells.

### 3.6 miR‐18a inhibition decreases cell viability and miR‐18a expression predicts poor patient survival in MM

**Figure 7:**
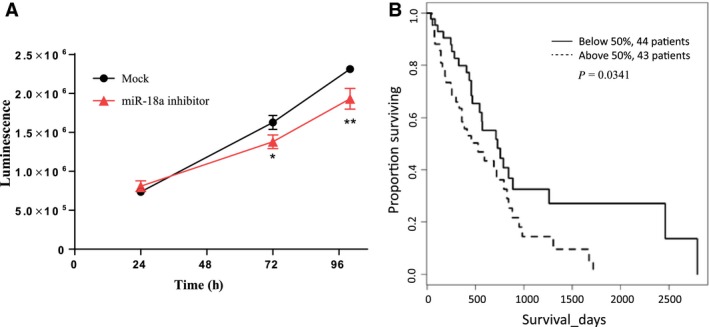
Cell viability after miR‐18a inhibition and survival analysis of miR‐18a in MM. (A) Cell viability measured by CellTiter‐Glo Luminescence assay 24, 72, and 100 h after transfection of miR‐18a inhibitors into H2452 MM cells *P =* *0.027 at 72 h and **P =* *0.0092 at 100 h comparing miR‐18a inhibitor group to its matching mock control. Error bars represent mean ± SD (*n* = 3), and *P* values were calculated using a multiple *t*‐test. (B) Kaplan–Meier curve for survival analysis based on TCGA dataset. MM patients (*N *=* *87) were divided into two groups depending on their miR‐18a expression level: one group (*N* = 43) with miR‐18a expression above the median level and the other group (*N* = 44) with miR‐18a expression below median level.

Previously, we have shown that PIAS3 is a tumor suppressor in MM cells and that low PIAS3 protein expression predicted poor survival in MM patients (Dabir *et al*., 2014). Because our results here support a role for miR‐18a in the regulation of PIAS3 protein expression in MM cell lines, we investigated its potential role as a therapeutic target in MM cells by inhibiting miR‐18a in cells and by determining its clinical importance in MM patient survival using the TCGA database (https://cancergenome.nih.gov/). As shown in Fig. 7A, miR‐18a inhibition significantly decreased cell viability compared to the mock control after 72 h (*P *=* *0.027) and 100 h (*P *=* *0.0092) in H2452 MM cells. Complementing this result, survival analysis (shown in Fig. 7B) suggests that higher expression of miR‐18a significantly correlates with poor patient survival outcome (*P = *0.0341). In contrast, none of the other miRs in the same miR‐17‐92 cluster with miR‐18a had a similar significant correlation with survival (shown in Fig. S2). These results suggest that targeting miR‐18a may benefit patient survival in MM.

## 4 Discussion

A previous study by our laboratory showed that as a tumor suppressor, low PIAS3 expression predicts poor patient survival in MM, yet the mechanism(s) regulating PIAS3 expression in MM still remain unclear. Examination of the TCGA database in cBioPortal revealed that PIAS3 rarely undergoes genetic alteration in MM (1.2%). We have also previously shown that in NSCLC cell lines PIAS3 is not regulated at an epigenetic level, adding to the uncertainly of the mechanism of PIAS3 regulation (Kluge *et al*., 2011). Here, we show that in MM cell lines, PIAS3 protein expression is broadly downregulated, while PIAS3 mRNA levels remain largely constant, indicating a post‐transcriptional regulation of PIAS3 protein expression.

One major mechanism of post‐transcriptional regulation is protein degradation via the proteasome pathway. Early studies in glioblastoma multiforme (GBM) and breast cancer have indicated that in tumor cells derived from these cancers, PIAS3 expression is suppressed by proteasomal degradation (Borghouts *et al*., 2010; Brantley *et al*., 2008). Other studies have reported a tri‐partite motif‐containing protein 8 (TRIM8) mediated PIAS3 ubiquitination in patient‐derived GBM neurosphere cells and PIAS3‐transfected HEK293 cells (Okumura *et al*., 2010; Zhang *et al*., 2017a). Moreover, nitric oxide (NO) has also been shown to accelerate PIAS3 protein degradation through the ubiquitination pathway in HeLa cells (Qu *et al*., 2007). To explore the possibility of protein degradation regulating PIAS3 expression in MM, we investigated the effect of proteasome inhibitor (MG‐132 and bortezomib) treatment on three MM cell lines with low endogenous PIAS3 expression. In contrast with the results of previous studies, we could show no increase in PIAS3 protein, suggesting that post‐translational regulation does not play a major role in suppressing PIAS3 protein expression in MM. Further experiments with CHX demonstrated the remarkable stability of PIAS3 protein.

Protein expression can also be regulated at the level of mRNA translation. miRs represent one class of translational regulators that are frequently reported to be highly dysregulated in multiple malignancies. In fact, several miRs, including miR‐18a, have been reported to regulate PIAS3 expression in other diseases, including gastric cancer, multiple myeloma, and chronic lymphocytic leukemia (Brock *et al*., 2011; Carabia *et al*., 2017; Huang *et al*., 2016; Pitari *et al*., 2015; Wang *et al*., 2017; Wu *et al*., 2013; Xiong *et al*., 2012). What distinguishes our study is that we used an unbiased miR microarray screen coupled with bioinformatic analysis to identify miR‐18a as a potential effector of PIAS3 downregulation. Although we could not directly show with cultured cells that transient overexpression of miR‐18a mimics downregulates PIAS3 levels by western blotting, likely due to the high PIAS3 protein stability shown in CHX assays, we could demonstrate specific downregulation by miR‐18a using PIAS3 3′UTR luciferase reporter assays as a surrogate for endogenous PIAS3 expression. Moreover, endogenous PIAS3 expression was markedly increased by miR‐18a inhibition with cultured cells, confirming its regulatory role on PIAS3 expression. In addition, by conducting survival analysis using the TCGA database, our study is the first to reveal that miR‐18a expression is negatively correlated with patient survival outcome in MM, which is consistent with our previous data in MM showing a positive correlation between PIAS3 protein expression and patient survival outcome (Dabir *et al*., 2014).

Much of the attention focused on miR‐18a stems from it being a member of the miR‐17‐92 cluster, which was one of the first tumorigenic clusters identified (Zhang *et al*., 2017b). A previous study in MM has shown that the miR‐17‐92 cluster is globally upregulated in MM cells compared to normal tissue (Balatti *et al*., 2011). Here, our results show that the survival significance in MM was unique to miR‐18a, as none of the other miRs in the same cluster had a similar negative correlation with survival (see Fig. S2). Two other studies in MM generating miR microarray profiles also suggest that miR‐18a is significantly upregulated in MM cell lines and MM tumor tissue compared to normal tissue (Amatya *et al*., 2016; Ramirez‐Salazar *et al*., 2014). Although none of these studies identified any key downstream targets of miR‐18a, they do support our idea that miR‐18a plays an important role in MM.

According to our previous study, overexpression of PIAS3 has been shown to inhibit cellular growth in MM cell lines (Dabir *et al*., 2014). We and others have also reported that restoring PIAS3 expression by curcumin and other natural compounds, such as ascochlorin and brassinin, have anti‐tumor effects, indicating that PIAS3 restoration can be a potential therapeutic strategy for cancer treatment (Dabir *et al*., 2014; Dai *et al*., 2015; Lee *et al*., 2015; Saydmohammed *et al*., 2010). As a negative regulator on PIAS3 expression, miR‐18a represents a potential target to increase PIAS3 expression. In this regard, a class of chemically engineered anti‐sense oligonucleotides called ‘anti‐miRNA’, also known as ‘antagomirs’, have been synthesized as miRNA inhibitors to target ‘oncomiRs’ both in cancer cell lines and in animal models (Ishida and Selaru, 2013; Krutzfeldt *et al*., 2005; Stenvang *et al*., 2012). Excitingly, these newly developed treatments have also been tested on animal models and in clinical trials (Janssen *et al*., 2013; Leone *et al*., 2013; Sicard *et al*., 2013; Wagenaar *et al*., 2015), making miRNA‐based therapeutic strategies a promising way to treat different diseases, including human cancers. Because our miR inhibition assay and survival analysis have identified miR‐18a as a potential oncogenic miR in MM, targeting miR‐18a with antagomirs may represent a novel approach to reduce future mortality in MM.

## 5 Conclusions

In summary, our study has investigated potential post‐transcriptional regulators of PIAS3 expression in MM. In contrast with results of previous studies, we observed no regulatory role for protein degradation in PIAS3 expression; however, we did find that miR‐18a may play a role to downregulate PIAS3 expression at a translational level by binding to the PIAS3 3′UTR. Furthermore, inhibition of miR‐18a restored PIAS3 expression and was associated with decreased cell viability. Taken together, miR‐18a may represent a promising therapeutic target in MM.

## Author contributions

TH, KM, NS, GW, and AD conceived and designed the project. TH and KM performed the experiments and analyzed the data. YC performed the survival analysis. TH and GW wrote the article. AD supervised the project.

## Supporting information


**Fig. S1.** PIAS3 protein levels in HAY and YOU MM cell lines.
**Fig. S2.** Survival curves for other miRs in cluster 17‐92.

**Table S1.** List of 687 miR candidates from microarray screening.
