# Supplementary material for: Post‐transcriptional regulation of PIAS3 expression by miR‐18a in malignant mesothelioma
Source: Mol Oncol. 2018 Oct 23;12(12):2124–35. doi: 10.1002/1878-0261.12386 (PMC6275277; doi:10.1002/1878-0261.12386)
Supplement: Supplementary file 1 — Fig. S1. PIAS3 protein levels in HAY and YOU MM cell lines. Fig. S2. Survival curves for other miRs in cluster 17‐92. [file MOL2-12-2124-s001.pptx]

## Slide 1
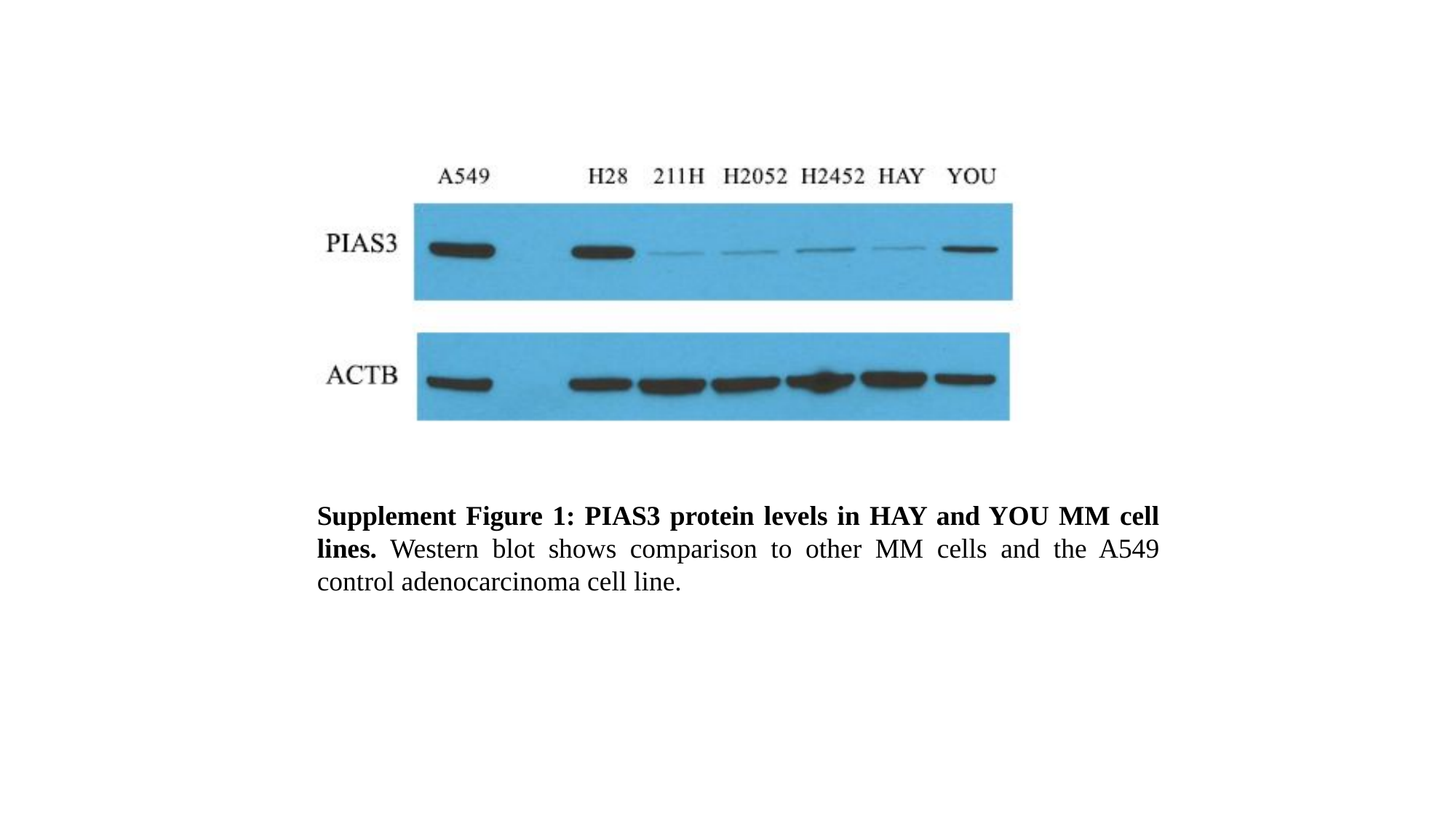

Supplement Figure 1: PIAS3 protein levels in HAY and YOU MM cell lines. Western blot shows comparison to other MM cells and the A549 control adenocarcinoma cell line.

## Slide 2
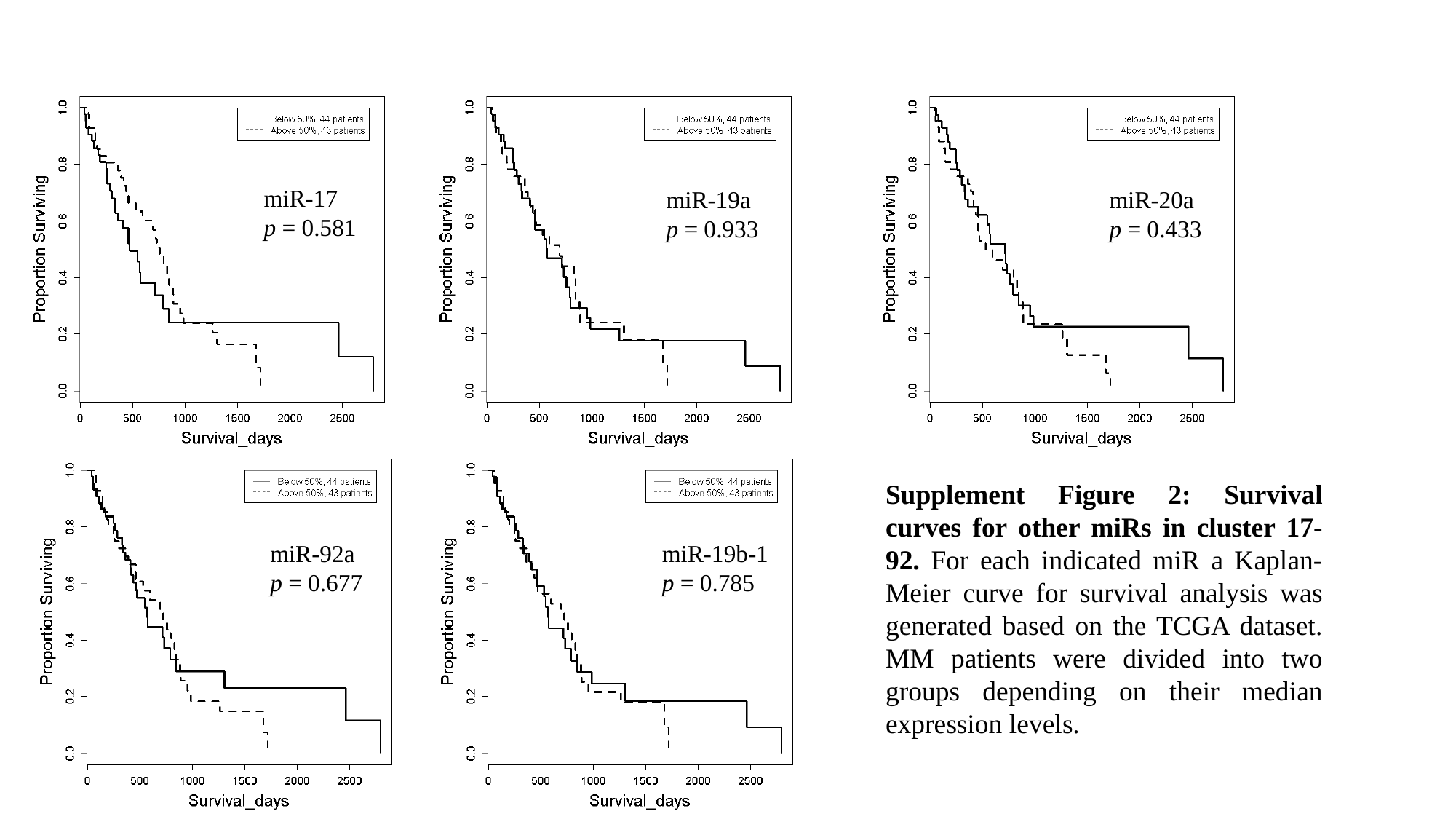

miR-19a
p = 0.933
miR-20a
p = 0.433
miR-19b-1
p = 0.785
miR-92a
p = 0.677
Supplement Figure 2: Survival curves for other miRs in cluster 17-92. For each indicated miR a Kaplan-Meier curve for survival analysis was generated based on the TCGA dataset. MM patients were divided into two groups depending on their median expression levels.
miR-17
p = 0.581
miR-17
p = 0.581
